# Arctigenin: pharmacology, total synthesis, and progress in structure modification

**DOI:** 10.1080/14756366.2022.2115035

**Published:** 2022-09-12

**Authors:** Dan Wu, Lili Jin, Xing Huang, Hao Deng, Qing-kun Shen, Zhe-shan Quan, Changhao Zhang, Hong-Yan Guo

**Affiliations:** Key Laboratory of Natural Medicines of the Changbai Mountain, Affifiliated Ministry of Education, College of Pharmacy, Yanbian University, Jilin, China

**Keywords:** Arctigenin, pharmacology, total synthesis, structure modification

## Abstract

*Arctium lappa L.* is a prevalent medicinal herb and a health supplement that is commonly used in Asia. Over the last few decades, the bioactive component arctigenin has attracted the attention of researchers because of its anti-inflammatory, antioxidant, immunomodulatory, multiple sclerosis fighting, antitumor, and anti-leukemia properties. After summarising the research and literature on arctigenin, this study outlines the current status of research on pharmacological activity, total synthesis, and structural modification of arctigenin. The purpose of this study is to assist academics in obtaining a more comprehensive understanding of the research progress on arctigenin and to provide constructive suggestions for further investigation of this useful molecule.

## Introduction

Natural products are components or metabolites found in animals, plants, insects, marine organisms and microorganisms. They are very significance for the development of new drugs. With the emergence of human genome sequencing and high-throughput screening technology, the molecular targets of compounds isolated from traditional drugs have been confirmed, which provides exciting possibilities for researchers to develop new molecular entities to treat diseases^1–3^.

Newman et al. reported that from 1946 to 2019 mentioned 1881 drugs, about 441 (23.5%) came directly or indirectly from natural products. Furthermore, 40 (53.3%) of the 75 small molecules in the field of anti-tumour were natural products from 1946 to 1980^4^. The research of natural products is extensive and it shows excellent performance. For example, both xanthohumol and curcumin have anti-tumour activity^5–6^.

Natural products are a treasure house worthy of attention, and have great potential in the development of new drugs because of their rich sources. Arctigenin, a phenylpropane dibenzylbutyrolactone lignan isolated from Forsythia koreana, Saussurea heteromalla, Wikstroemia indica, Centaurea diluta, has been previously reported^7^. In the past research, arctigenin have made significant progress in determining the development of lead compounds for the treatment of human diseases and the research of related molecular mechanisms. Therefore, this article combines 75 pharmacological activities and 85 chemical related articles. Briefly reviews the pharmacology and total synthesis of arctigenin, and reviews the medicinal chemistry research progress of new derivatives of arctigenin, in order to understand the therapeutic potential and value of arctigenin.

## The chemical constituents of *Arctium lappa L*

The compounds isolated from *Arctium lappa L.* generally contain lignin, volatile oil, fatty acids, and terpenoids. The lignin is the highest in terms of content.

### Lignans

**Figure 1.**
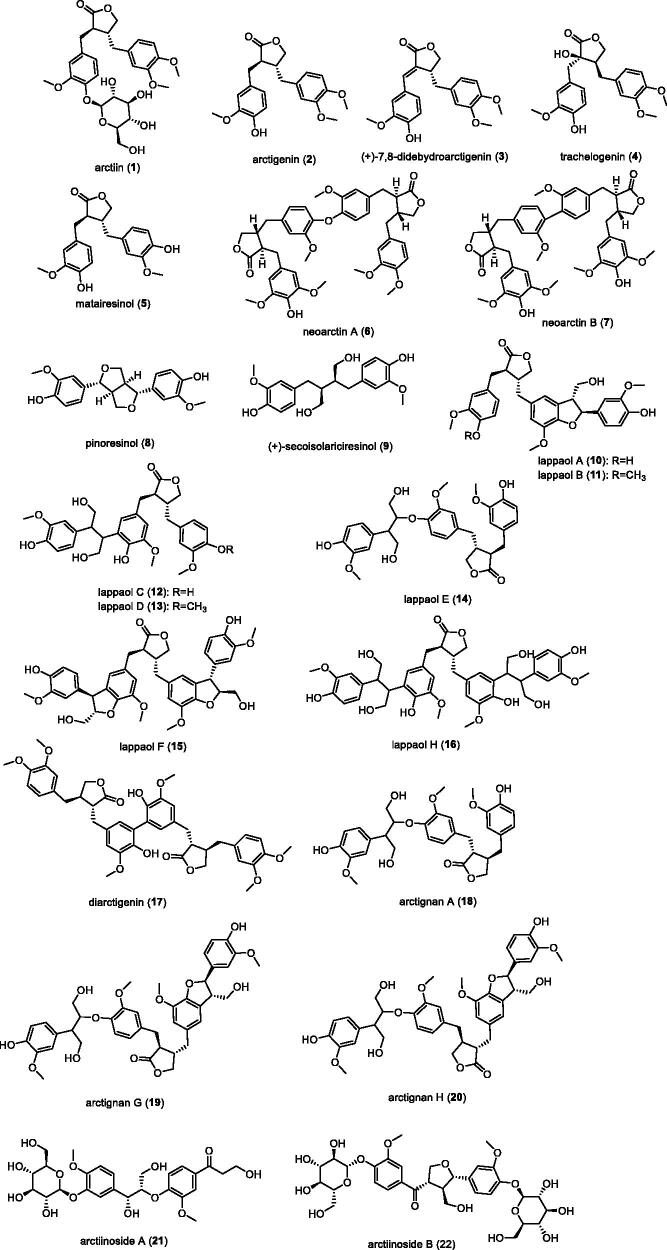
The structure of lignin compounds in *Arctium lappa L.*

Lignin mainly includes: arctiin (**1**)^8^, arctigenin (**2**)^8^, (+)-7,8-didebydroarctigenin (**3**)^8^, trachelogenin (**4**)^9^, matairesinol (**5**)^10^, neoarctin A (**6**)^11^, neoarctin B (**7**)^12^, pinoresinol (**8**)^13^, (+)-secoisolariciresinol (**9**)^13^, lappaol A (**10**)^14^, lappaol B (**11**)^15^, lappaol C (**12**)^15^, lappaol D (**13**)^16^, lappaol E(**14**)^17^, lappaol F (**15**)^18^, lappaol H (**16**)^18^, diarctigenin (**17**)^19^, arctignan A (**18**)^11^^,^^20^, arctignan G (**19**)^11^, arctignan H (**20**)^11^, arctiinoside A (**21**)^21^, arctiinoside B (**22**)^21^ et al. The structural formulas of these compounds are shown in the Figure 1.

### Volatile oil components

*Arctium lappa L.* contains a variety of oil components. Zhao et al. analysed the oil components of arctigenin using GC-MS and chemometric resolution methods, and performed qualitative and quantitative analyses to identify the major components linalool (0.6%), camphor (1%), and p-menthone (0.1%)^22^.

### Terpenoids

Terpenoids, mainly sesquiterpenoids triterpenoids and tretaterpene, were isolated from *Arctium lappa L.* Among them, sesquiterpenoids mainly include eremophilene, fukinone, petasitolone, β-eudesmol, dehydrofukinone^23^ and arctiol and β-cineole^24^; the triterpenoids mainly include taraxasterol, taraxasterol palmitate, and taraxasterol acetate^23^ and β-sitosterol^25^ and the tretaterpene contains β-carotene^25^.

### Fatty acids

*Arctium lappa L.* contains large amounts of fatty oils, the majority of which are fatty acids such as methyl palmitate, methyl linoleate, methyl linoleate, and linoleic acid^15^.

### Other ingredients

*Arctium lappa L.* also contains flavonoids, phenolic acids, and a few alkaloids^15^.

## The pharmacological activity of arctigenin

Fructus Arctii is the dry and ripe fruit of *Arctium lappa L.*, a biennial herb of the Compositae family. *Arctium lappa L.* is one of the traditional Chinese Medicine commonly used in clinic, and its leaves and roots can also be used. Traditionally, it is used for dispelling wind and clearing heat, dispersing lung and resolving phlegm, promoting pharynx and promoting body fluid, detoxification and detumescence^26^. *Arctium lappa L.* has the functions of antibacterial, antioxidant, hypoglycaemic, lipid-lowering, vasodilation, improvement of atherosclerosis, anti-tumour, anti-mutagenesis, and immune regulation, and has potential application value^27^.

**Figure 2.**
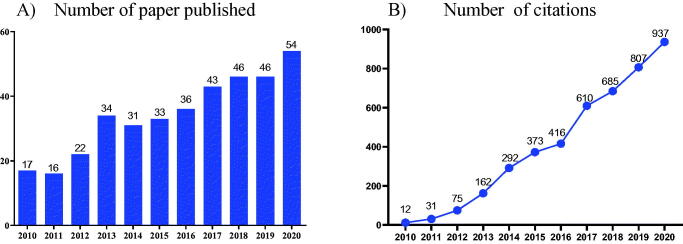
**(**A) Number of papers published between 2010 and 2020 containing the keyword "arctigenin", searched according to Web of Science. (B) Citations between 2010 and 2020 using the keyword "arctigenin", searched according to Web of Science.

Arctigenin and its glycoside, arctiin, are the two main active components of *Arctium lappa L.*^28^. Moreover, arctigenin is an important bioactive component of Fructus Arctii with good anti-inflammatory, anti-tumour, antioxidant effects and other therapeutic effects, attracting more and more attention in recent years (Figure 2).

### Anti-tumour activity

Arctigenin has anti-tumour effect against a variety of cancers such as pancreatic cancer, gastric cancer, colon cancer, and liver cancer. Details about the anti-cancer activity of arctigenin and its mechanism of action are given in this section.

#### Arctigenin inhibits tumour cell proliferation

The activities of arctiin and arctigenin differ with respect to the treatment of liver cancer. In an experimental study of hepatocellular carcinoma, Hirose et al.^29^ observed very low therapeutic effect of arctiin against liver cancer, while arctigenin had a remarkable anti-hepatocellular carcinoma effect. The toxic effects that arctiin and arctigenin have on human hepatocellular carcinoma HepG2 cells were tested *in vitro* by Moritani et al.^30^; the results indicated that both inhibit the proliferation of HepG2 cells while having little effect on normal hepatocytes.

Arctigenin upregulates the expression of Bax while downregulates the expression of Bcl-2; moreover, it significantly inhibited the proliferation of SNU-1 and AGS in a time- and dose-dependent manner^31^. Takasaki administered arctiin and arctigenin topically and orally, and found that both had a significant inhibitory effect on skin cancer cells^32^.

#### Arctigenin promotes apoptosis

Arctigenin inhibited the PI3K/Akt signaling^33–34^, thereby significantly inhibiting the viability of liver cancer cells in a concentration-dependent manner. The molecule also induces apoptosis, activates caspase-9 and caspase-3, and reduces the expression of Bcl-xL, MCL1, and survivin and the phosphorylation of mTOR and S6K, which can be reversed by the overexpression of constitutively active Akt. Arctigenin can inhibit colon and rectal cancer by inducing the apoptosis of colon and rectal cancer cells, as confirmed by Hausott et al^35^.

Arctigenin has been found to selectively destroy the oxidative phosphorylated human pancreatic carcinoma cell line PANC-1. This selective killing was due to induction of apoptosis and apoptotic necrosis through endoplasmic reticulum stress, mitochondrial membrane permeabilization, and cysteine activation^36^. Additionally, arctigenin has the ability to eradicate cancer cells. Experiments have shown that arctigenin inhibited the growth of pancreatic tumours in nude mice and suppressed the growth of pancreatic cancer cell lines^37^.

#### Arctigenin regulates the cell cycle

Study by Yang et al. indicated that the inhibitory effect of arctigenin on the activity of bladder cancer T24 cells was dependent on dose and time. Arctigenin reduced the expression of cyclin D1 and arrested tumour cells in the G1 phase, but did not affect the expression levels of CDK4 and CDK6. In addition, arctigenin selectively altered phosphorylation of members of the MAPK superfamily, significantly reduced the phosphorylation of ERK1/2, and activated the phosphorylation of p38^38^.

#### Arctigenin inhibits cancer cell metastasis

Arctigenin inhibited metastasis in human breast cancer cells by inhibiting AP-1 transcriptional activity through the ERK1/2 and JNK1/2 MAPK pathways, without affecting the p38 MAPK pathway. The molecule also inhibited matrix metalloproteinase-9 and urokinase-type plasminogen activator through the AKT, NF-κB, and MAPK signalling pathways to exert anti-metastatic effect in breast cancer, regardless of the ER expression^29^.

Study by Lee et al. demonstrated the anti-colon and rectal cancer activity of arctigenin. They demonstrated that arctigenin inhibited the proliferation of SW480 cells, induced apoptosis, and also significantly inhibited the invasion and metastatic ability of highly metastatic SW480 cells. It has also been observed that apoptosis was induced by a decrease in the expression of anti-apoptotic Bcl-2 and transcriptional activator (IAP-1), while the expression of the pro-apoptotic genes Smax and Bax increased in the presence of the molecule^35^^,^^39^^,^^40^.

#### Summary of arctigenin anti-tumour activity

Arctigenin has unique anti-tumour activity. Several studies have demonstrated the inhibitory effect of arctigenin against various tumours, both *in vitro* and *in vivo*. In summary, arctigenin is a potential antitumor natural product and is expected to provide new drug candidates for cancer treatment. Table 1 summarises a number of *in vitro* and *in vivo* cancer studies that used arctigenin.

**Table 1.** Summary of *in vitro* and *in vivo* studies with arctigenin against various cancer.

Cancer	*In vitro* studies	*In vivo* studies	Potential molecular mechanisms	Ref.
Hepatocellular	IC_50_ (Hep G2 cells) = 38.29 μM (12 h), 1.99 μM (24 h), 0.24 μM (48 h)	–^b^	Activate caspase-9 and caspase-3, Bcl-2↓, Bax↑, release of cytochrome c, deactivation of PI3K/p-Akt, P53↑, Fas/FasL↑, NF-κB↓	[^41^]
	IC_50_ (Hep G2 cells) = 30 μM (48 h), IC_50_ (Hep3B cells) = 40 μM (48 h)	–^b^	Apoptosis, Activate caspase-9 and caspase-3, Bcl-xl↓, Mcl-1↓, survivin↓, phosphorylation of mTOR and S6K↓,	[^42^]
Hepatoblastoma	IC_50_ (HUH-6 cells) = 4 μM (48 h)	–^b^	Apoptosis, Caspase-3/7↑, Caspase-8↑	[^43^]
Colon Cancer	CT26, MC38, and SW620 cells^a^	50 mg/kg/day, tumour nodules↓, tumour formation↓, metastasis↓	cell cycle arrest and apoptosis, E-cadherin↑, mesenchymal markers↓, N-cadherin↓, vimentin↓, β-catenin↓, Snail↓, MMP-2↓, MMP-9↓	[^44^]
	IC_50_ (SW480 cells) = 42.5 μM (48 h)	–^b^	β-catenin↓, cyclin D1↓	[^45^]
	IC_50_ (HCT116 cells) = 0.82 μM (48 h)	zebrafish treated with arctigenin (5 μM or 15 μM), inhibited angiogenesis	N-cadherin↓, β-catenin↓, vimentin↓, VEGF↓, MMP-2↓, MMP-9↓, N-cadherin↑	[^46^]
Prostate Cancer	LNCaP, LAPC-4, and WPE1-NA22 cells^a^	50 and 100 mg/kg, tumour growth↓, tumour volume↓, tumour weight↓	VEGF↓, FGFb↓, Bax/Bcl-2↑, EGF↓, PDGF-BB↓, NGF-b↓, TNF-α	[^47^]
Lung Adenocarcinoma	A549^a^	–^b^	NPAT↓, cyclin E/CDK2↓, cyclin H/CDK7↓, Bax↑, Fas, Caspase-3, 8,9↑, Akt-1↓, Bcl-2↑, Bad↓	[^48^]
Gastric Cancer	SNU-1 and AGS cells^a^	–^b^	Bcl-2↓, Bax↑, cyclin D1↓, cyclin E↓, CDK4↓, CDK2↓, P15↑, P21↑, p-Rb (ser 780)↓	[^31^]
Bladder Cancer	T24 cells^a^	–^b^	cyclin D1↓, phospho-ERK1/2↓, induce phospho-p38	[^38^]
Breast Cancer	MCF-7 cells^a^	–^b^	mTOR pathway↓, leading to autophagy-induced cell death, ERα↓, LC3-II/LC3-I↑	[^49^]
	MCF-7 and MDA-MB-231 cells^a^	–^b^	MMP-9↓, activated Akt and NF-κB, activate MAPKs, ERK 1/2, JNK1/2 and p38, AP-1 transcription↓	[^50^]

^a^Arctigenin inhibited the growth of tumour cells, but did not give IC_50_.

^b^Not available.

^↓^Reduce, inhibition or down-regulated.

^↑^Increase or up-regulated.

### Anti-leukemia activity

Hirano T et al. evaluated cytotoxicity and anti-leukemic activity of both arctiin and arctigenin using HL-60 (human blood cancer cells) and four anticancer drugs as positive controls. The results showed that arctigenin significantly inhibited the proliferation of HL-60 cells (IC_50_ < 100 ng/mL) and that its anti-leukemic activity was comparable to that of anticancer drugs used in clinical practice^51^.

### Anti-inflammatory effect

Arctigenin exerts favourable anti-inflammatory activity. Extensive pharmacological research has shown that is can impart anti-inflammatory effect in conditions such as edoema, colitis, and acute lung injury^52^.

Even low concentrations (<32 μM/L) of arctigenin have been found to significantly inhibit TNF-α in RAW264.7 mouse macrophages and U937 human macrophages induced by lipopolysaccharides, with no toxic effect on normal cells. TNF-α inhibitors are known to have a beneficial effect on inflammation. This conclusion was made through *in vitro* study by Cho et al.^53^.

Arctigenin has also been found to inhibit the nuclear transcription factor receptor activator κB ligand (RANKL)-mediated differentiation of osteoclasts (mouse bone marrow macrophages), suggesting beneficial effects of arctigenin in treating rheumatoid arthritis and osteoporosis^54^. The related mechanism may involve inhibitory effect of arctigenin on production of the inflammatory stimuli NO, TNF-α, and IL-6, with no effects observed on the expression and activity of cyclooxygenase-2. It also inhibited excess NO production by downregulating the inducible nitric oxide synthase (iNOS) expression and enzyme activity in lipopolysaccharide-stimulated macrophages^55^, and exerted an anti-inflammatory effect by effectively inhibiting the MKK (inhibition of TNF-α), MAP kinase, and AP-1 activation^56^.

Arctigenin exerts its anti-inflammatory effect through its antioxidant activity and inhibition of the STAT signalling. It has been shown to significantly reduce the phosphorylation of STAT1, STAT3, and JAK2 in lipopolysaccharide-stimulated RAW264.7 cells and prevent translocation of STAT1 and STAT3 to the nucleus, thereby suppressing the expression of iNOS and inhibiting the expression of inflammation-related genes such as IL-1β, IL-6, and MCP-1, which contain the promoters of the STAT components. It has also been found to suppress the expression of iNOS in macrophages by inhibiting the JAK-STAT signalling pathway^57^.

The arctigenin-associated degradation of iNOS in lipopolysaccharide-stimulated mouse macrophage-like RAW 264.7 cells was due to CHIP-associated ubiquitination, which is proteasome-dependent process. It also reduces iNOS phosphorylation and subsequently inhibits the enzymatic activity of iNOS by inhibiting the ERK and Src activation, and promotes ubiquitination and iNOS degradation following lipopolysaccharide stimulation. In conclusion, burdock seed sapogenins promote degradation of iNOS through the CHIP-related proteasome pathway and inhibit its enzymatic activity^40^.

Lee et al.^58^ showed that 0.1 μM/L of arctigenin caused 26.70 ± 4.61% decrease in COX-2 gene expression and 32.84 ± 6.51% decrease in prostaglandin E2 content. In fact, regarding the molecular mechanism by which arctigenin inhibits inflammation, most studies have demonstrated that it inhibits the activation of the NF-κB pathway by suppressing the lipopolysaccharide-induced p65 nuclear translocation and IκBα, MAPK, and PI3K phosphorylation in cells^33^^,^^59^. NF-κB and MAPK signalling pathways have been identified as the main targets of anti-inflammatory action of arctigenin in several *in vivo* inflammation models^60–61^ (Table 2).

**Table 2.** summarises a number of *in vitro* and *in vivo* studies that have investigated the anti-inflammatory properties of arctigenin.

Model	*In vitro* studies	*In vivo* studies	Potential molecular mechanisms	Ref.
Acute Lung Injury	–^a^	lung wet-to-dry (W/D) ratio↓, MPO activity↓, TNF-α↓, IL-1β↓, IL-6↓	pIκBα↓, NF-κB p65↓, phosphorylation of AMPKα↓, phosphorylation of AMPKα↑	[^60^]
	–^a^	50 mg/kg, lung injury↓, TNF-α↓, IL-6↓, MIP-2↓, NO↓	iNOS↓, HO-1↑, ERK↓, p38↓, JNK↓	[^61^]
	–^a^	10, 20 and 40 mg/kg, IL-8↓, TNF-α↓, IL-1β↓, MOP↓, lung wet-to-dry (W/D) ratio↓	PI3K/Akt phosphorylation↓, NF-κB↓	[^62^]
LPS stimulation	RAW264.7, NO↓	–^a^	iNOS↓, ERK and Src activation↓	[^40^]
	RAW264.7, IL–1β↓, IL–6↓, MCP–1↓	–^a^	iNOS↓, COX-2↓, phosphorylation of STAT1, STAT3↓, JAK2↓	[^57^]
Liver injury from acute hepatitis	–^a^	ALT↓, AST↓, TNF-α↓, IFN-γ↓, IL-17A↓, IL-17F↓, IL-1β↓, CXCL10↓, TGF-β1↑, IL-4↑	inhibition of pro-inflammatory cytokines and chemokines, mediator of macrophages	[^63^]

^a^Not available.

^↓^Reduce, inhibition or down-regulated.

^↑^Increase or up-regulated.

### Anti-colitis effect

Arctigenin ameliorated inflammation in ulcerative colitis by inhibiting the PI3K/Akt pathway and polarising the M1-type macrophages to produce M2-like macrophages both *in vitro* and *in vivo*^33^, and it is the main active ingredient that reduced the dextran sodium sulfate-induced inflammatory response in mouse colon^64^. It has also shown protective effects in a rat gastric ulcer model^65^. Table 3 summarises several *in vivo* studies that evaluated anti-colitis action of arctigenin.

**Table 3.** Summary of *in vivo* studies on the anti-colitis activity of arctigenin.

Model	*In vivo* studies	Potential molecular mechanisms	Ref.
DSS induction	50 mg/kg, DAI↓, MPO↓, IL-6↓, TNF-α↓, MIP-2↓, MCP-1↓, MAdCAM-1↓, ICAM-1↓, VCAM-1↓, E-selectin↓, MDA↓, SOD↑, GSH↑	phosphorylation of p38 MAPK, ERK, JNK, IκBα and p65↓,	[^64^]
	the differentiation of Th1 cells↓, IFN-γ↓, IL-17A↓, IL-17F↓, IL-21↓, IL-22↓, Th1↓, Th17↓	phosphorylation of genes p70S6K and RPS6↓, *via* mTORC1 pathway	[^66^]
TNBS induce	30 and 60 mg/kg, TNF-α↓, IL-1β↓, IL-6↓,	PI3K↓, AKT↓, NF-κB↓	[^33^]

^↓^Reduce, inhibition or down-regulated.

^↑^Increase or up-regulated.

### Antiviral effect

An *in vivo* study investigated anti-human immunodeficiency virus (HIV-1) activity of arctigenin and found that arctigenin can inhibit the viral response of HIV-1-infected human cell lines^67^. Arctigenin generally acts in the integration stage and has an inhibitory effect on integration of proviral DNA into its own cells. Moreover, arctigenin significantly inhibited the expression of HIV-1 virus *in vitro*, particularly *via* expression of the anti-HIV core protein P17 and the anti-HIV matrix protein P24^68^.

Hayashi et al.^69^ demonstrated through *in vitro* experiments that arctigenin has the ability to interfere with the early replication of influenza A virus and has an inhibitory effect on the release of progeny viruses. The study also showed that arctigenin did not increase the drug resistance of the virus, while the control drug oseltamivir induced drug resistance, reducing the therapeutic efficacy of the anti-viral drug to 50%. This finding revealed the huge potential of this molecule and had a profound impact on the development of new drugs using arctigenin. The antiviral activity of arctigenin on porcine circovirus type 2 (PCV2) was found similar to that of ribavirin. Therefore, arctigenin may also protect against PCV2 infection^70^. Arctigenin also showed antiviral activity in EPC cells against SCVC, a fish rhabdovirus^71^.

### Vascular protective effect

Arctigenin can upregulate the three proteins ABCA1, ABCG1, and apoE, which promote THP-1 macrophages through activation of the PPARγ and liver X receptor α (PPAR-γ/LXR-α) signalling pathways, promoting cholesterol efflux. This is an important mechanism that prevents the occurrence of atherosclerosis^72^. The PI3K/Akt signalling pathway can also induce endothelial nitric oxide synthase (NOS) and inhibit the cerebral vasospasm caused by subarachnoid haemorrhage in rats^73^.

### Protection against memory problems and brain damage

Arctigenin has been used as a neuroprotective agent for the treatment of epidemic encephalitis B by inhibiting glutamate transmission and reducing the induced field response^74–75^. The results of studies indicate that in addition to its effect on super-excited neurons under physiological conditions, arctigenin can cross the blood-brain barrier and interact with acid-sensitive ionotropic glutamate receptors in the brain. These results suggest that arctigenin is a potentially useful new pharmacological method that can inhibit the glutamate-induced response of the central nervous system *in vivo*; that is, it can reduce the response of somatosensory cortical neurons^76^. Arctigenin has also been shown to effectively improve memory impairment by eliminating target amyloid in AD mouse models^77^. Arctigenin can treat brain injury that occurs after needle insertion by anti-inflammatory and anti-apoptotic mechanisms^78^, can protect rats with focal cerebral ischaemia reperfusion by inhibiting neuroinflammation^79^, and can regulate the PI3K/Akt signalling pathway by increasing the expression of the haem oxygenase-1 gene in rat primary astrocytes^80^.

### Hepatoprotective effect

The proliferation of hepatic stellate cells (HSC) is an important factor in the process of fibrotic liver injury. Through G0/G1 cell cycle arrest, arctigenin has been found to induce continuous p27Kip1 induction by interfering with the PI3K/Akt/FOXO3a signalling pathway, inhibiting the proliferation of activated hepatic stellate cells^81^.

### Anti-insect activity

A study conducted in 2009 found that arctigenin isolated from the traditional Chinese medicine burdock by the activity tracking method has anti-insect activity and can kill the medium-sized finger ringworm (*Dactylogyrus intermedius*) in fish. The median effective concentration was 0.62 mg/L (EC_50_) after 48 h, which was significantly lower than that of the control drug mebendazole (1.25 mg/L)^82^. The study also showed that arctigenin has anthelmintic activity against the third generation of Kobayashi, a fish parasite. A study has shown that 4.00 mg/L arctigenin can kill 100% of the third-generation insects infecting goldfish in 4 h *in vivo*, with EC_50_ values of 1.85 mg/L and 1.58 mg/L after 24 h and 48 h, respectively^83^. Another study found that a crude extract of burdock fruit has schistosomiasis-killing activity, with arctigenin as the main active ingredient that was believed to be responsible for the activity^84^.

### Other pharmacological effects

Studies have shown that the binding effect of platelet-activating factor can also be inhibited by lignan, the main component of burdock seed. It is believed that the main active ingredient that inhibits platelet aggregation is arctigenin, suggesting it as an effective drug against platelet activating factor^85^.

Zhao et al. found that the peroxidation of liposomes and apoptosis of cardiomyocytes can be inhibited by arctigenin, along with the elimination of free radicals associated with cardiomyocytes^86–87^.

Arctigenin has also been found to act as an oestrogen receptor β selective agonist that limits mTORC1 activation and the subsequent Th17 cell differentiation^88^. As a potential anti-arrhythmic lead compound, arctigenin also inhibits arrhythmia resulting from aconitine through regulation of multiple ion channels^86^, and relieves endoplasmic reticulum stress by activating the adenosine monophosphate-activated protein kinase (AMPK), thereby preventing and treating various diseases^89^. It has also been shown to improve the swimming endurance of sedentary rats by regulating the antioxidant pathways^75^.

Arctigenin and (–)-pinoresinol are human thyroid hormone receptor β antagonists^90^, and arctigenin has an inhibitory effect on melanin synthesis by tyrosinase^91^. Arctigenin is a new inhibitor of heat shock response in mammalian cells^92^. It exhibited a neuroprotective effect by upregulating the expression of mouse primary neurons P-CREB and human SH-SY5Y neuroblastoma cells^93^.

Yang et al.^94^ found that arctigenin regulates the cardiovascular system by inhibiting the influx of calcium ions and the release of internal calcium, thereby exhibiting a relaxing effect on isolated guinea pig trachea and pulmonary artery, and rat trachea and thoracic aorta. In addition, cell-level studies have found that 3 μM arctigenin can significantly promote the uptake of glucose by the L6 skeletal muscle cells, thereby exhibiting anti-diabetic activity^95^. Ishihara et al.^92^ found that arctigenin hinders the production of heat shock response proteins, resulting in cells with little heat resistance, thereby regulating the heat shock response.

### Pharmacological effects of arctigenin in combination with other drugs

Combining arctigenin with the glucose analog 2-deoxyglucose has been found more conducive in killing tumour cells and has few toxic side effects^96^. The combination of arctigenin with green tea polyphenols and curcumin has also been shown to enhance the preventive effect on prostate cancer and breast cancer cells^97^. Combining it with quercetin resulted in synergistic increase in anti-proliferative effect against prostate cancer cells^98^.

## Total synthesis of arctigenin and its analogues

The natural product arctigenin possesses good biological activity and acts as a lead compound. The main laboratory methods that have been utilised for the production of arctigenin are silica gel column chromatography purification^37^^,^^99^, organic reagent extraction^55^^,^^69^, centrifugal partition chromatography^100^, and chemical combination^101^, chemical combination^101^. However, all these methods have shortcomings such as low extraction efficiency, cumbersome steps, and necessity of laboratory equipments. The low content of active ingredients in herbal medicines and reliance on isolated extraction to obtain sufficient amounts of compounds not only renders production costly, but also leads to a series of problems related to sustainable use of natural medicinal resources and environmental sustainability. Therefore, artificial synthesis of natural products with excellent activity is an important means to solve the above problems. Current studies have shown that the technology for the total synthesis of arctigenin is readily available^102^.

### Synthesis of (–)-arctigenin and its enantiomer

**Figure 3.**
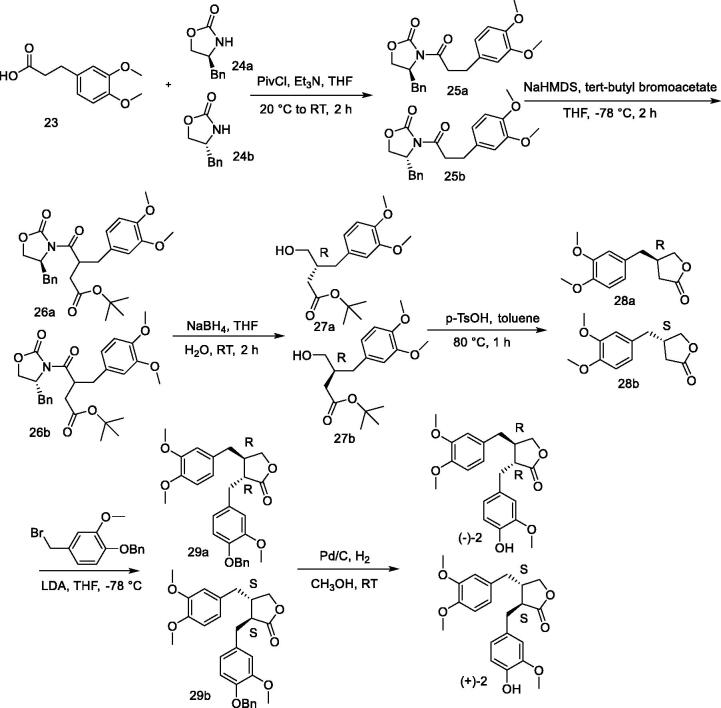
A new method for asymmetric synthesis of arctigenin.

A new method for the asymmetric synthesis of (–)-arctigenin is shown in Figure 3^103^. Using 3,4-dimethoxyphenylpropionic acid (**23**) as the starting material and the chiral prosthetic group 4-benzyloxazolidine ketone (**24**), which was condensed to obtain compound **25**, and then using a large hindered organic base, sodium bis(trimethylsilyl) amide (NaHMDS), a carbonyl α-alkylation reaction was performed to obtain compound **26**, which was then reduced to eliminate the chiral auxiliary to obtain alcohol **27**. The ester exchange reactions that were catalysed by p-toluenesulfonic acid produced butyrolactones **28a** and **28b** with ee values of 98% and 96%, respectively; this was followed by introduction of the derivatized benzyl group at the α-position of the ester carbonyl group of 8 using the large-site resistive organic base lithium diisopropylamine (LDA) to obtain **29a/29b** (de > 99%), and finally Pd/C-catalysed hydrogenolysis was carried out to remove 58% and 55% of the phenolic hydroxyl protecting group, respectively, *via* six steps. The total yield of 97% and ee value of 96% were obtained for both (–)-arctigenin and (+)-arctigenin.

### Enantioselective synthesis: (–)-Arctigenin, and (–)-isoarctigenin (35)

**Figure 4.**
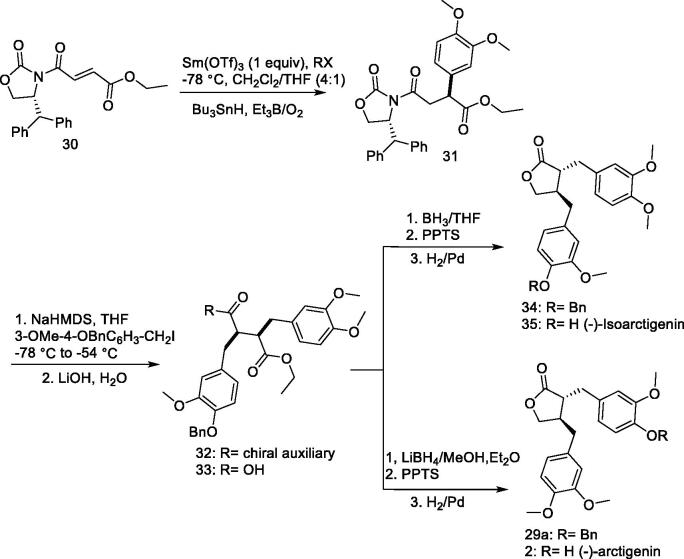
Synthesis of (–)-isoarctigenin (**35**) and (–)-arctigenin.

A new method involving highly regioselective and stereoselective addition of radicals to asymmetric fumarates was reported by Sibi et al.^104^ to exploit the synthesis of lignin natural products from common intermediates (Figure 4). Compound **30** was used as the starting material. The reaction was completed by adding benzyl **30** to CH_2_Cl_2_/THF in the presence of Sm(OTf)_3_ at −78 °C to obtain product **31.** The required natural product was finalised by adding a second benzyl chloride solution. Consequently, 3-methoxybenzyl bromide was added after treating **31** with an equivalent amount of NaHMDS (1:1) at −78 °C for 1.25 h to give intermediate product **32**, which was then cleaved effortlessly to obtain excellent yield of the key intermediate **33** using LiOH/H_2_O_2_/THF. The conversion of **33** to **34** consists of selective carboxyl reduction and lactonization. Reduction *via* debenzylation produced (–)-isoarctigenin (**35**). The reduction of compound **31** to **29a** (76% in two steps) was achieved by alkylation with 3,4-dimethoxybenzyl iodide (64%). Debenzylation was then utilised to produce (–)-arctigenin.

### Highly enantioselective total synthesis of natural Lignan lactones

**Figure 5.**
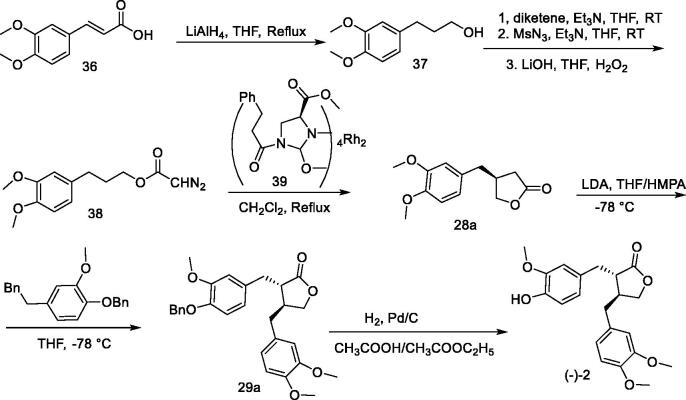
Synthesis of arctigenin from compound 3,4-dimethoxycinnamic acid.

Bode et al.^105^ pointed out that it is necessary to develop a catalytic method for domain and enantiomer control that can be applied to the total synthesis of natural lignans such as arctigenin. The synthesis of (–)-arctigenin can thus be achieved *via* a nine-step reaction using 3,4-dimethoxycinnamic acid (**36**) **(**Figure 5**)**. The reaction involved reducing 3,4-dimethoxycinnamic acid to hydroxyl with lithium aluminium hydride to obtain 3,4-dimethoxyphenylpropanol **37**, and the corresponding diazoacetate **38** was obtained by one-pot method. The key lactone intermediate **28a** was thus prepared using catalyst **39** at 94% ee in 62% isolated yield. Compound **28a** was alkylated with 4-(benzyloxy)-3-methoxybenzyl bromide and prepared from 4-hydroxy-3-methoxybenzyl alcohol *via* two steps to give disubstituted γ-lactone **29a**. Hydrogenolysis of the benzyl group was then conducted to give (–)-arctigenin with an optical purity of 94%.

### Synthesis of four stereoisomers of arctigenin

**Figure 6.**
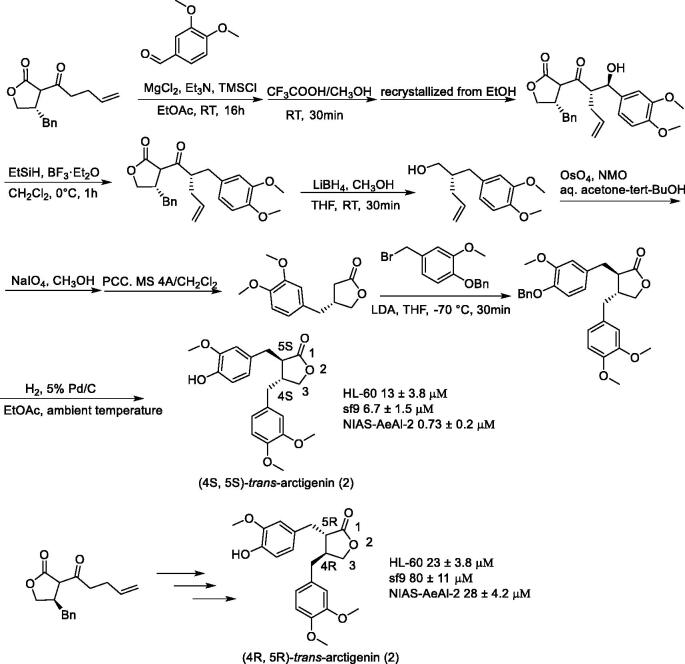
Synthesis of (4S, 5S)-*trans*-arctigenin and (4 R, 5 R)-*trans*-arctigenin.

**Figure 7.**
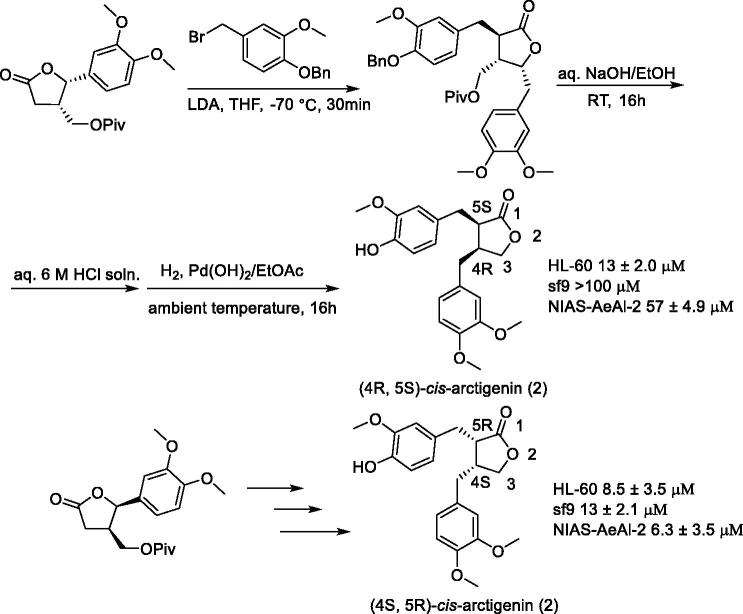
Synthesis of (4 R, 5S)-*cis*-arctigenin and (4S, 5 R)-*cis*-arctigenin.

(4R, 5 R)-(–)-*trans*-arctigenin^106^ is a butyrolactone-type lignan with two chiral centres; it has a wide range of biological activities. Anti-aggregation activity of (4S, 5S)-(+)-*trans*-arctigenin^107^ and cAMP phosphodiesterase inhibitory activity of cis-arctigenin^108^ have been reported. However, no studies comparing the biological activities of all stereoisomers to elucidate the stereochemical effects of the two chiral carbons have been carried out. Yamauchi et al.^109^ synthesised four stereoisomers of arctigenin and compared their biological activities (Figure 6 and 7). Further, they prepared derivatives with various substituents on the aromatic ring, and then proved the biological activities of these derivatives.

**Figure 8.**
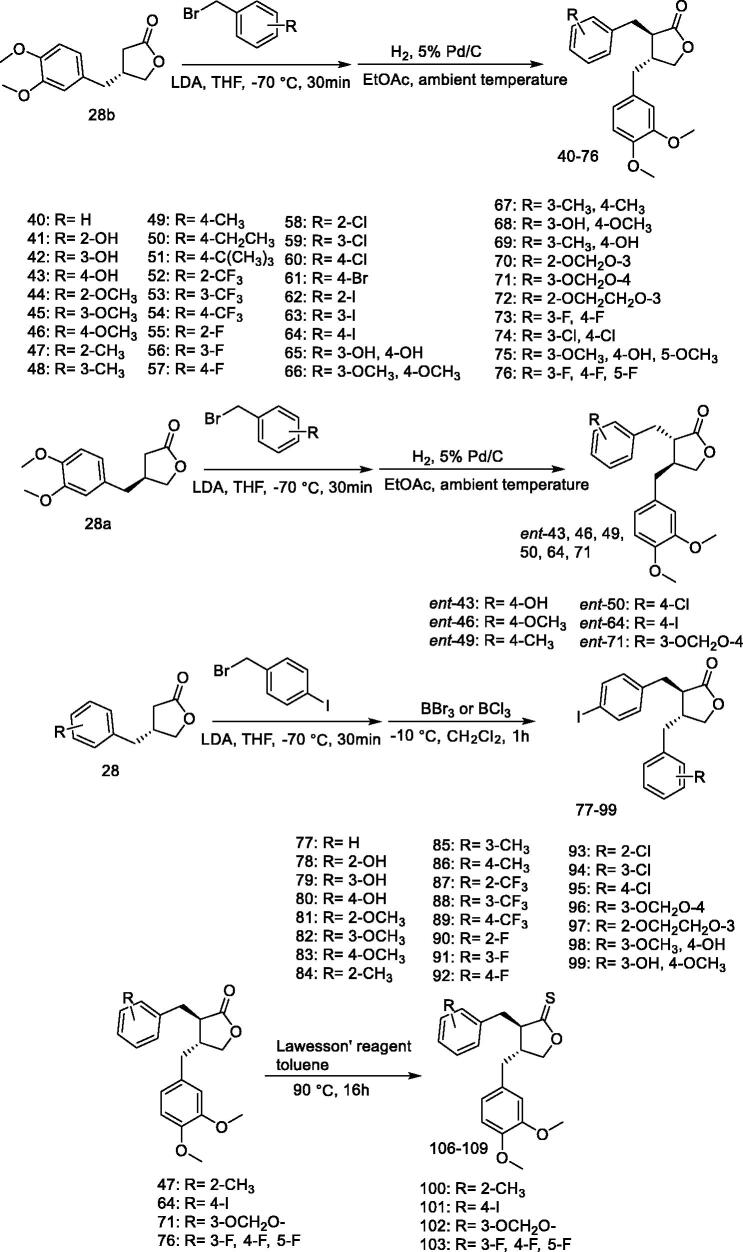
Syntheses of 7-aryl-3′,4′dimethoxy derivatives **40**–**99** and *ent-***43**, **46**, **49**, **50**, **64**, **71,** and thionolactones **100–103**.

Arctigenin exhibits stereospecific cytotoxicity against insect cells. One of the stereoisomers of arctigenin, (4 R, 5 R)-*trans*-arctigenin, shows stereospecific cytotoxicity against insect cells, Sf9, and NIAS-AeAl-2 cells. Yamauchi et al.^109^ synthesised the arctigenin derivatives **40–103** (Figure 8) and found that compounds **63**, **64**, and **71** are at the same level as (4 R, 5 R)-*trans*-arctigenin. Examination of the thionine derivatives indicated that compounds **101** and **142** have similar activity levels to that of (4 R, 5 R)-*trans*-arctigenin, which was found to increase the expression of the 28S rRNA gene in the ribosomes of Sf9 cells, however, DNA degradation was not observed.

### *Synthesis of (–)-arctigenin derivatives ent-*41–43, *ent-*44–46, *ent-*66, *ent-*68, 104–115

Type 2 diabetes is a metabolic disorder that is characterised by high glucose levels, insulin resistance, and impaired insulin secretion^110^. Several studies have shown that reduced glucose uptake by skeletal muscle leads to type 2 diabetes and associated metabolic syndrome^111–113^. Therefore, therapies that regulate glucose uptake are promising strategies for the treatment of metabolic disorders^95^.

**Figure 9.**
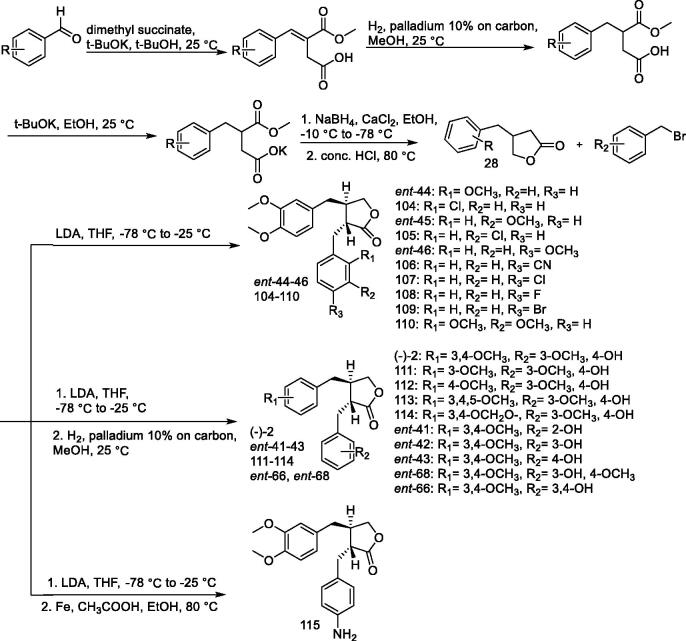
Synthesis of compounds *ent*-**41–46**, *ent*-**66**, *ent*-**68**, and **104–115**.

Duan et al. prepared analogs of the natural product (as activators of AMPK) arctigenin (*ent-***41–43,** *ent-***44–46,** *ent-***66,** *ent-***68, 104–115**) to evaluate their effects on 2-deoxyglucose uptake in L6 myotubes and their possible use in ameliorating metabolic disorders (Figure 9). Racemic (–)-arctigenin was found to display enhanced uptake similar to that of (–)-arctigenin. The results suggest that the substitution of the para-hydroxyl group on the benzene ring of the C2 benzyl portion of the arctigenin skeleton by chlorine produces a substance **107**, which exhibits excellent uptake activity and helps in avoiding possible metabolic problems. Compound **107** stimulated glucose uptake and fatty acid oxidation *via* AMPK activation *in vitro* The chronic administration of **107** has been shown to reduce blood glucose levels and improve lipid metabolism in ob/ob mice^114^.

## Structural modification of arctigenin

Arctigenin has excellent biological activity and therefore potential for further development as a lead compound. As the pharmacological effects of arctigenin have been confirmed, the structural modification of arctigenin has become increasingly popular. The shortcomings of arctigenin include insufficient activity, poor solubility, and low bioavailability. The structural modification of arctigenin is performed mainly to improve its biological activity, solubility, and metabolic ability so as to obtain new derivatives with greater developmental value. The following studies introduce the theoretical research based on the structural modification of arctigenin.

### Synthesis of pyrimidine derivatives of arctigenin

**Figure 10.**
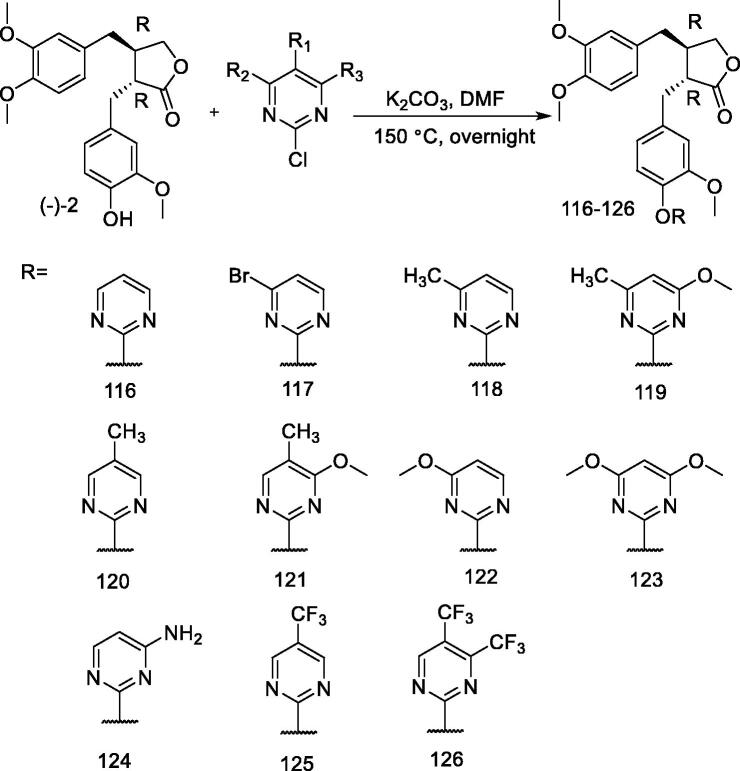
Synthesis of pyrimidine derivatives of arctigenin (**116–126**).

A series of pyrimidine derivatives of arctigenin were designed and synthesised by Wang et al.^103^. The key intermediates that were required to replace 2-chloropyrimidine have been synthesised by multiple synthetic routes, and arctigenin derivatives (**116–126**) have been synthesised by linking arctigenin with these intermediates *via* ether bonds **(**Figure 10**)**.

### Arctigenin and its analogues are oxidised with PIFA

Phenyliodine(III) bis(trifluoroacetate) (PIFA) reacts with the phenols in methanol to produce cyclohexadienone and quinone ketal, which are valuable intermediates in organic synthesis^115–116^. The oxidation of phenol in less nucleophilic solvents such as acetonitrile or trifluoroethanol (TFE) allows intramolecular reactions that lead to cyclization^117–118^. The most promising approach is the realisation of carbon-carbon bond formation^119^. Ward attempted to use this reaction to simulate the oxidation-induced cyclisation involved in the biosynthesis of various lignans and the synthesis of several compounds^120^.

**Figure 11.**
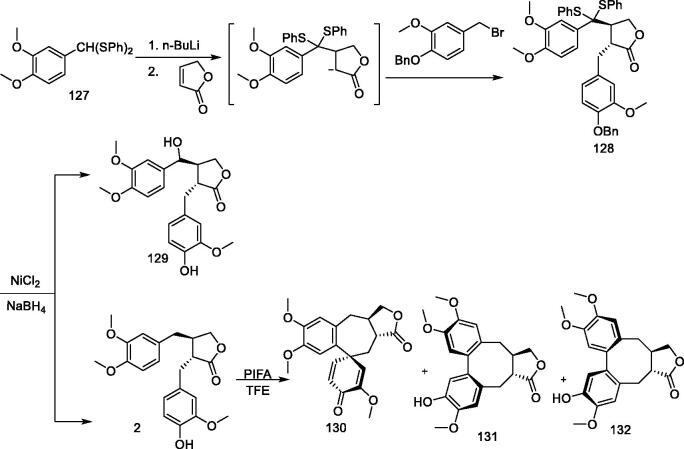
Arcigenin is treated with PIFA.

3,4-Dimethoxybenzaldehyde bis(phenylthio)acetal (**127**) was lithiated, followed by the addition of butenolactone and the subsequent capture of the enolates with the corresponding benzyl bromide to give compound **128** with a 55% yield. After debenzylation, arctigenin was obtained at a yield of 68% to produce hydroxyl derivative **129** with a 20% yield (Figure 11**).** Arctigenin was also treated with PIFA (1.2 equiv.) in TFE for 24 h to obtain a mixture of products from which two major fractions were isolated; **130** with a yield of 13% and a combination of **131** and **132**, with an overall yield of 14%.

### Synthesis of N-acyl sulfamate from arctigenin

**Figure 12.**
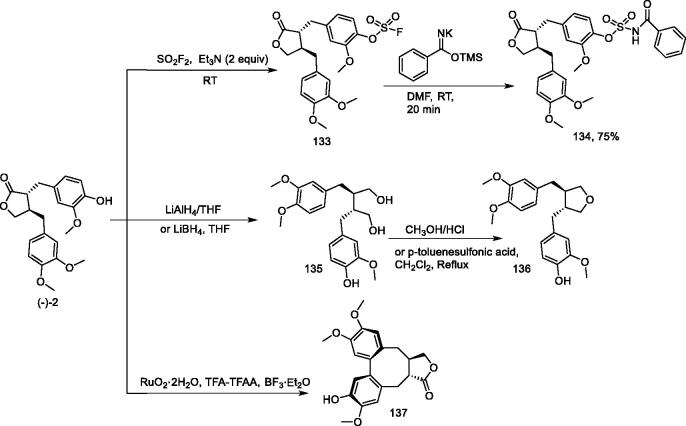
Synthesis of compounds **133–137**.

Zhang et al.^121^ reported an efficient and simple method for the synthesis of N-acylaminosulfonates from fluorosulfonates and potassium trimethylsiloximate as amide precursors. This method produced a wide range of substrates under mild and base-free reaction conditions and short reaction times, with high to excellent yields. Zhang et al. applied this method to arctigenin (Figure 12) by reacting the phenolic hydroxyl group with sulphuryl fluoride (SO_2_F_2_) in anhydrous N,N-dimethylformamide (DMF) in the presence of triethylamine for 3 h to produce the corresponding fluorosulfate **133**, which was then reacted with trimethylsiloxyimide in DMF for 20 min at room temperature to obtain N-acyl sulfamate **134** (75%).

### Synthesis of 3,4-dibenzyltetrahydrofuran skeleton

Ward and Eich et al.^122–123^ synthesised a series of trans-3,4-dibenzyltetrahydrofurans using ruthenium tetrafluoroacetate (trifluoroacetic acid) *via* oxidative cyclisation to obtain high yields of dibenzocyclooctadiene lignans of the isostane series. To construct the 3,4-dibenzyltetrahydrofuran skeletal structure, arctigenin was first reduced to 1,4-dibenzylbutanediol **135** and then dehydrated to give compound **136** (Figure 12).

### Oxidative cyclisation of 2,3-dibenzylbutyrolactones using ruthenium tetra(trifluoroacetate)

Ward et al.^124^ used a series of *cis*- and *trans*-2,3-dibenzylbutyl lactones, which were oxidatively cyclized using tetrakis (trifluoroacetic acid) ruthenium to give dibenzocyclooctadiene lactones.

Arctigenin was treated with tetrakis(fluoroacetic acid-acetic acid)ruthenium, which was obtained from two equivalents of RuO_2_•2H_2_O in a TFA-TFAA mixture containing traces of BF_3_•Et_2_O. The mixture was stirred at room temperature for 24 h to give a single product (**137**) with an 80% yield (Figure 12).

### Anti-tumour activity of arctigenin derivatives

#### Synthesis of (–)-arctigenin derivatives 138–147 by Cai et al

**Figure 13.**
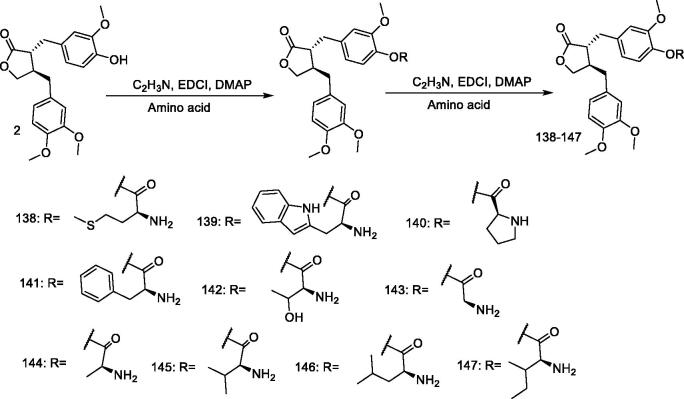
Synthesis of compounds **138–147**.

Cai et al.^125–126^ synthesised amino acid derivatives by reacting five amino acids with arctigenin using tert-butoxycarbonyl as a protecting group (Figure 13). The results showed that amino acid derivatives without protecting groups had better water solubility and nitrite scavenging ability than those with protecting groups. The ability of the derivatives to scavenge nitrite was significantly higher than that of arctigenin. Based on these results, **138, 141, 145** and **147** were selected at a dose of 40 mg/kg to evaluate their antitumor activity, and these compounds showed inhibition rates of 55.87%, 51.40%, 69.27%, and 43.58%, respectively. The results indicate that the compounds **138, 141, 145** and **147** have strong antitumor activity both *in vitro* and *in vivo*, and can improve the immune response of tumour-bearing mice.

#### Compound 145 treats myelosuppression

Cai et al.^125–126^ used chemical methods to modify the structure of arctigenin and synthesise a series of arctigenin amino acid ester derivatives. The dissolution and *in vitro* and *in vivo* pharmacological activities of arctigenin valine ester (**145**) were investigated. The compound has a simple process of synthesis, high yield, strong water solubility, significantly enhanced pharmacological activity, and significantly improved oral bioavailability, and is metabolised by the body to act as a technical drug. Han et al.^127^ therefore speculated that **145** can be used as a drug candidate for the treatment of the myelosuppression caused by chemotherapy. After taking **145**, the peripheral blood cells of mice were observed to gradually return to normal, the number of bone marrow nucleated cells increased, the thymus index increased, the spleen index decreased, the number of haematopoietic progenitor cells increased, and the number of haematopoietic cytokines decreased. Compound **145** promoted the transformation of myelosuppressive cells from G0/G1 to S phase and from S to G2/M phase. Compound **145** can upregulate the expression of MEK and p-ERK, low-dose **145** was not as effective as high-dose in all aspects. In summary, **145** can effectively relieve the myelosuppression caused by the intraperitoneal injection of CTX 100 mg/kg, can promote the proliferation and differentiation of haematopoietic progenitor cells, and can improve immunity.

In the early stage, han et al.^127^ modified the structure of arctigenin and synthesised a series of amino acid ester derivatives of arctigenin. The solubility, *in vitro* and in vivo pharmacological activities of arctigenin were investigated, and arctigenin valine ester was screened. Arctigenin valine ester has the advantages of simple process, high yield, strong water solubility, significantly enhanced pharmacological activity and significantly improved oral bioavailability; And metabolise in the body to play the role of technical drugs.

#### Synthesis of (–)-arctigenin derivatives 148–153 by Chen et al

**Figure 14.**
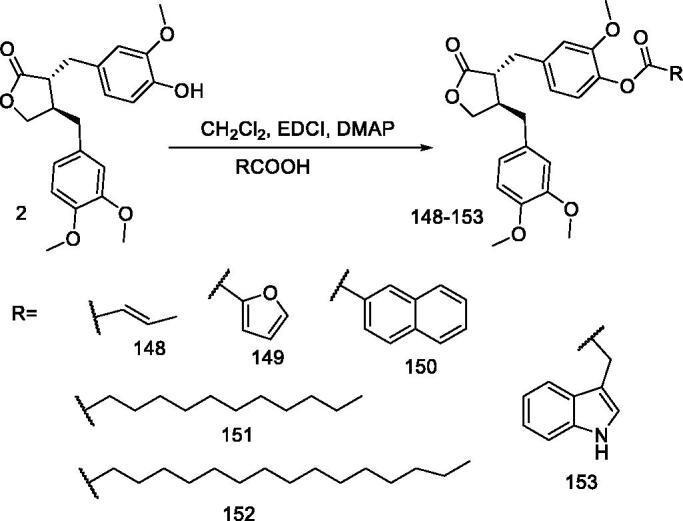
Synthesis of compounds **148–153**.

Chen et al.^128^ reacted arctigenin with carboxylic acids (crotonic acid, furoic acid, 2-naphthoic acid, and indole-3-acetic acid), EDCI, and DMAP in dichloromethane under reflux at 60 °C to obtain six new monoester derivatives (**148–153**) (Figure 14). The properties of these derivatives were investigated using *in vitro* nitrite scavenging assay. The *in vivo* antitumor activity of the β-indole acetate ester of arctigenin (**153**) was studied at 20 and 40 mg/kg doses, with results showing that **153** had stronger antitumor activity in H22-bearing mice than that of arctigenin.

#### *Synthesis of (–)-arctigenin derivatives* 154–166

Solid tumours are generally associated with hypoxia, glucose starvation, and malnutrition due to insufficient vascular supply and/or the excessive demand of rapidly proliferating cells^129^. Glucose starvation has been shown to increase the invasive and metastatic potential of tumour cells, which is the main cause of cancer-related deaths^130^. In recent years, tumour cells have been reported to have the intrinsic ability to reduce the apoptotic potential by regulating their energy metabolism^131–132^. Therefore, selective targeting of tumour cells by inhibiting cellular energy metabolism under glucose starvation is an alternative strategy for antitumor therapy with minimal toxicity to normal tissues.

Arctigenin has been reported to exhibit antitumor effect in various xenograft models. Awale et al. reported that arctigenin is preferentially cytotoxic to cancer cells under conditions of glucose starvation^37^. Arctigenin has been shown to inhibit mitochondrial respiration under such conditions, leading to intracellular ATP depletion and ROS production, resulting in cell death^96^. Kudou et al. described the synthesis of arctigenin derivatives with variable modified O-alkyl groups and assessed their preferential cytotoxicity under glucose starvation; they revealed that the 4-hydroxy group of arctigenin is important for preferential cytotoxicity^37^^,^^133^.

**Figure 15.**
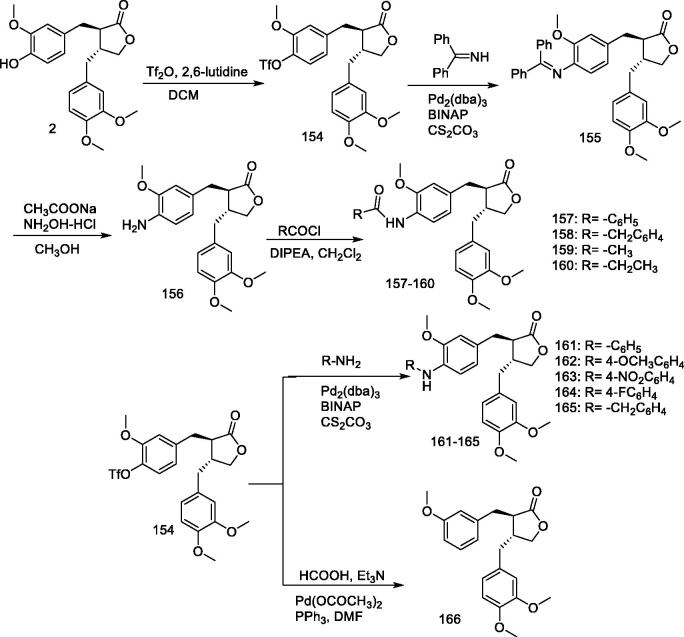
Synthesis of compounds **154–166**.

Owing to the important role of arctigenin 4-hydroxyl, Lei et al.^134^ designed and synthesised a series of 4-amino-4-dehydroxylan derivatives (**154–166**) (Figure 15) and evaluated their cytotoxicity against human A549 tumour cell line under sugar-deficient conditions. The results showed that 4-amino-4-dehydro xyloglucan was more cytotoxic than arctigenin and that addition of further substituents to the 4-amino group led to a significant decrease in cytotoxicity. Compound **156** showed the strongest cytotoxicity against A549 cell line, with an IC_50_ value of 2.85 μM, which is 2.3-fold that of arctigenin.

#### *(–)-Arctigenin derivatives* 167–172

Pancreatic cancer metastasises rapidly, leading to death within a short period of time after diagnosis. Consequently, patients with pancreatic cancer have the lowest 5-year survival rate among most cancers^135–136^. Pancreatic cancer is highly resistant to most known chemotherapeutic agents, including 5-fluorouracil and gemcitabine^137^; therefore, there is an urgent need for effective chemotherapeutic agents that can target pancreatic cancer.

**Figure 16.**
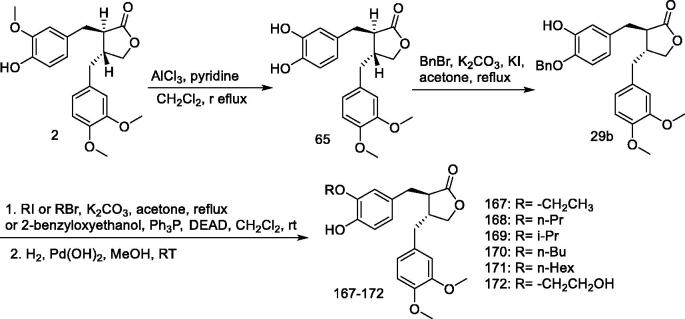
Synthesis of compounds **167–172**.

Kudou et al.^133^ synthesised arctigenin derivatives (**167–172**) with variable modified oxygen alkyl groups and evaluated their preferential cytotoxicity against human pancreatic cancer cell line PANC-1 under nutrient-deficient conditions (Figure 16). The results showed that the anticancer activity of **167–172** was weaker than that of (–)-arctigenin.

### Antiviral and antiparasitic activity

#### (–)-Arctigenin derivatives 173–208

The spring viremia of carp virus (SVCV) belonging to the genus Vesiculovirus in the family *Rhabdoviridae* is a bullet-shaped RNA virus that causes high mortality in common carp (*Cyprinus carpio*) and other fishes in the family *Cyprinidae*^138^. This virus also infects other fish such as Piscivora, catfish, snapper, salmon, and barb^139–140^. The extensive study of arctigenin that results from its broad range of biological activities led to recognition of its antiviral activity against SVCV in endothelial progenitor cells^71^^,^^141^^,^^142^. However, the practical application of arctigenin is limited because of its toxicity. Therefore, it is particularly important to structurally modify arctigenin to produce less toxic derivatives while maintaining its potency.

**Figure 17.**
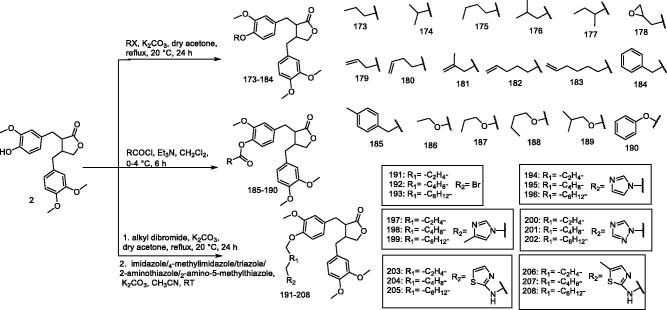
Synthesis of compounds **173–208**.

To search for anti-SVCV drugs, Chen et al.^143^ designed, synthesised, and evaluated several arctigenin derivatives (**173–208**) for their antiviral activity (Figure 17). The half-maximal inhibitory concentration (IC_50_) of 15 screened drug candidates (with a maximum inhibitory response of more than 90%) was compared in SVCV-infected Cyprinidae epithelioma cells, and compounds **189** and **196** showed IC_50_ values of 0.077 and 0.095 μg/mL, respectively. Further experiments showed that compounds **189** and **196** significantly reduced SVCV-induced apoptosis and had a protective effect on cell morphology 48 and 72 h post infection. In addition, **189** and **196** significantly inhibited the production of SVCV infection-induced reactive oxygen species, which was clearly observed in SVCV-infected cells. Based on these findings, **189** and **196** show promising application in the treatment of SVCV infection.

*Dactylogyrus intermedius* is a common ectoparasite that parasitises the gills of freshwater fish and represents the largest group of postnatal fish parasites. The parasite has a direct life cycle without an intermediate host and releases its eggs into the water to hatch, which then attach to the gills of the fish host^144^.

To control the parasitism of *D. intermedius*, Hu et al.^144^ designed, synthesised, and tested a new series of arctigenin derivatives. The anthelmintic activity of most derivatives was shown in the range 1–10 mg/L. Compared to the conventional drug praziquantel (EC_50_ = 2.69 mg/L), the ether derivatives **206** and **207** showed slightly higher antiparasitic activity with EC_50_ values of 2.48 and 1.52 mg/L, respectively. In addition, the arctigenin-imidazole hybrids **194** and **197** were also effective in removing intermediate entomopathogenic nematodes, with EC_50_ values of 2.13 and 2.07 mg/L, respectively. Structure-activity relationship analysis showed that the four-carbon linker and imidazole substituents can significantly improve the insect repellent activity and reduce the toxicity of the molecule. The above results indicate that **194** and **197** are considered to be promising lead compounds to prevent and control *D. intermedius* infection.

#### (–)-Arctigenin derivatives 191–199 and 209–227

Infectious haematopoietic necrosis virus (IHNV) is one of the three pop-up viruses listed by the World Organisation for Animal Health (OIE), which is causing serious losses in the aquaculture industry^145^. As a species pathogen that causes infectious haematopoietic organ necrosis (IHN), IHNV is highly pathogenic and widely transmissible, resulting in high mortality rates of 80–100% in salmonid species^146^. Therefore, there is an urgent need to develop an effective antiviral strategy to treat highly lethal IHNV. Studies have shown that arctigenin reduced the replication of carp virus (another fish elasmobranch virus) in spring viremia by 65%^71^.

**Figure 18.**
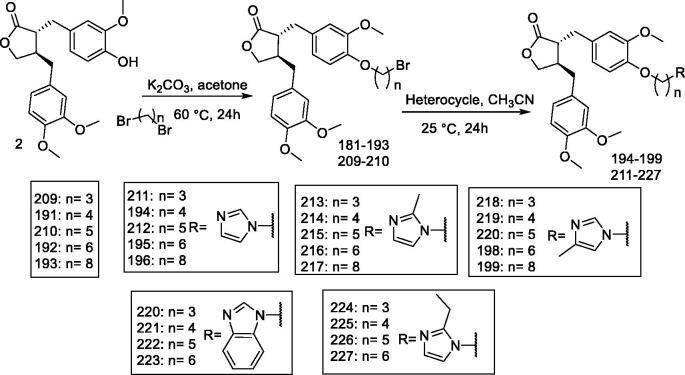
Synthesis of compounds **181–199** and **209–227**.

The results of a previous study in which a series of arctigenin derivatives (***191–199 and 209–227***) were synthesised to evaluate their antiviral activity against IHNV (Figure 18)^147^ indicated that the linker length and imidazole substituents play an important role in reducing IHNV replication. In this study, the arctigenin-imidazole hybrid derivative **217,** with a linker length of 8 carbons, reduced the replication of IHNV with an IC_50_ value of 1.3 μM. In addition, derivative **217** significantly inhibited IHNV-induced apoptosis and cell morphological damage. Mechanistically, the derivative **217** could not directly destroy viral particles. The addition time and virus binding assay revealed that the derivative **217** mainly affected the early replication of IHNV but did not interfere with the adsorption of IHNV. The derivative **217** can therefore be considered a promising drug for the treatment of IHNV.

#### (–)-Arctigenin derivatives 229–238 and 240–261

*Toxoplasmosis* is a global parasitic disease and is caused by the specialised intracellular parasite *Toxoplasma gondii*, which infects approximately one-third of the world’s population^148^. The traditional treatments for toxoplasmosis include ethidiazine, sulfadiazine, spiramycin, and atovaquone. Clinically, etanercept and sulfadiazine have shown significant anthelmintic effects, but the combination of the two drugs can cause serious adverse effects such as hypersensitivity reactions, bone marrow suppression, intolerance, and an increased risk of liver and renal complications^149^. To date, there is no ideal drug that can completely eradicate all forms of *Toxoplasma gondii*. Therefore, there is an urgent need to develop highly effective and less toxic tolerable drugs for the treatment of this parasitic infection.

**Figure 19.**
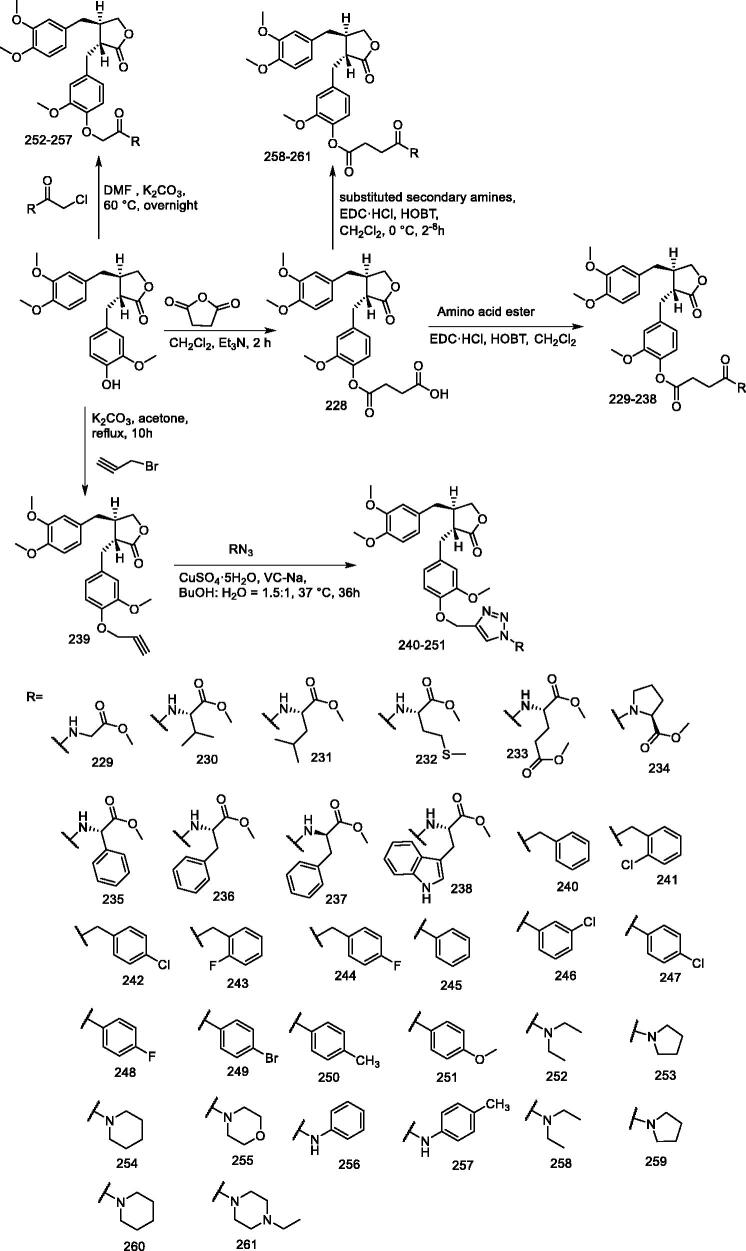
Synthesis of compounds **229–238** and **240–261**.

Zhang et al.^150^ designed and synthesised four new series of arctigenin derivatives (**229–238 and 240–261**) and evaluated their anti-*Toxoplasma gondii* activity both *in vitro* and *in vivo* (Figure 19). Among the synthesised compounds, compound **243** exhibited the strongest anti-Toxoplasma activity and the lowest cytotoxicity (*Toxoplasma gondii* IC_50_: 17.1 μM; IC_50_: ≥ 600.0 μM in HeLa cells; selectivity: 35.09), which was higher than that of both arctigenin (*Toxoplasma gondii* IC_50_: 586.4 μM; IC_50_: 572.7 μM in HeLa cells; selectivity: 0.98) and the positive control drug spiramycin (*Toxoplasma gondii* IC_50_: 262.2 μM; IC_50_: 189.0 μM in HeLa cells; selectivity: 0.72) for clinical application *in vitro*. In addition, compound **256** showed superior inhibition of Toxoplasma gondii *in vivo* to spiramycin. Compounds **243** not only significantly reduced the number of tachyzoites in the peritoneal cavity of mice, but also resulted in their partial malformation *in vivo* (*p* < 0.05). Compounds **243** therefore have the potential to be used as antiparasitic drugs and are valuable for further development.

### (–)-Arctigenin derivatives 149, 262–320

**Figure 20.**
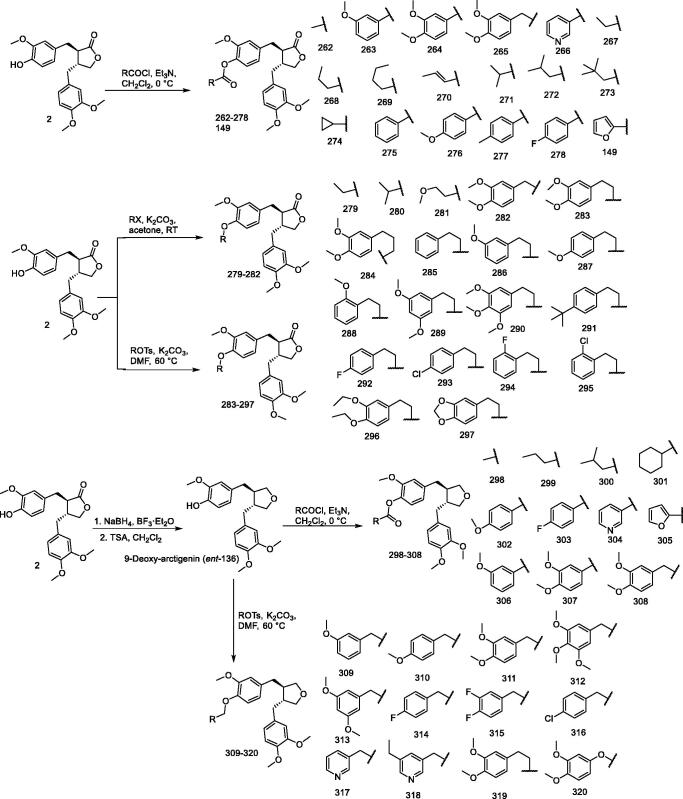
Synthesis of compounds **149, 262–320**.

AMPK, which is a heterotrimeric serine/threonine protein kinase, plays a crucial role in the regulation of systemic energy homeostasis^151–153^. As a cellular energy sensor, AMPK activation stimulates glucose uptake and fat oxidation while inhibiting adipogenesis and gluconeogenesis^154–156^. AMPK is therefore considered a potential therapeutic target for the treatment of obesity and type 2 diabetes^157–161^. Sida Shen et al.^162^ designed and synthesised a series of new arctigenin and 9-deoxy arctigenin derivatives (**149, 262–320**) with different esters and ether side chains at the phenolic hydroxyl position and evaluated their ability to activate AMPK effects in L6 myogenic cells. Preliminary biological evaluation showed that some alkyl ester and phenyl ether arctigenin derivatives showed potential activity in improving AMPK phosphorylation. Further conformational analysis showed that arctigenin derivatives **262, 269, 309, 311,** and **312** had superior ability to arctigenin regarding activation of AMPK (Figure 20). Arctigenin derivative **311** was identified as a promising lead compound exhibiting superior activity in AMPK activation.

## Conclusion

Natural products are a valuable source of bioactive molecules for drug discovery. *Arctium lappa L.* is a kind of Chinese herbal medicine, which has stable pharmacological effects in dispelling wind and heat, detoxification and swelling, and sore throat. Based on in-depth research on *Arctium lappa L.*, its components were isolated, and arctigenin was reported to be an important component.

**Figure 21.**
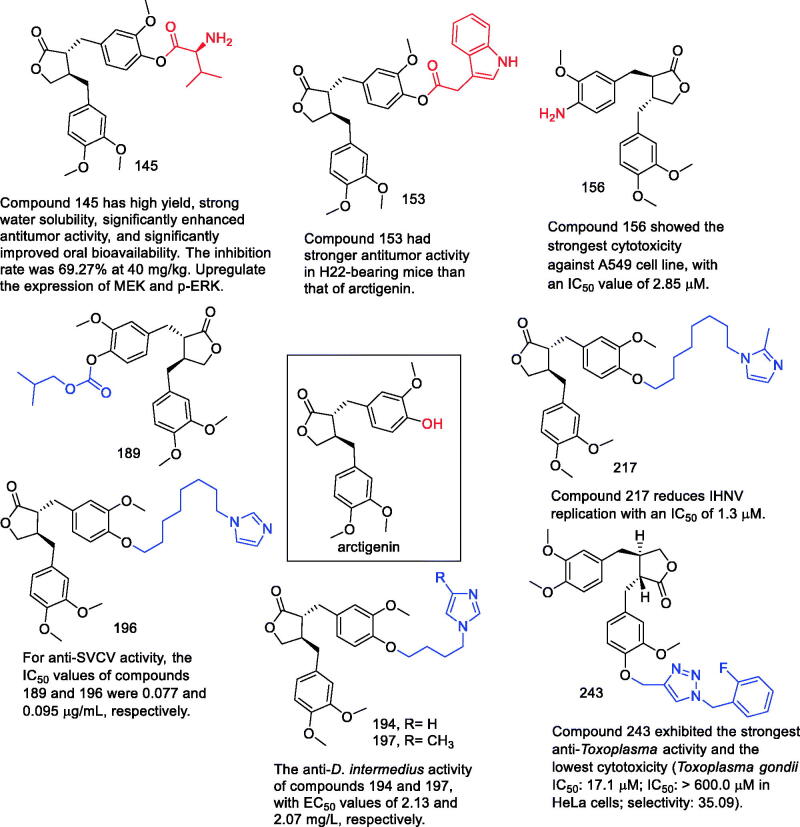
Graphical representation of the general structural anticancer and antiparasitic activity relationship of arctigenin derivatives.

Since the discovery of arctigenin, it has often been used as a lead compound for drug development because of its novel structure, strong pharmacological activity, and great developmental potential, with an increasing number of studies investigating the molecule. However, there are still some problems and new directions for future development in moving arctigenin to a viable therapeutic approach:

- Although arctigenin have received great attention in the past decade, their exact molecular mechanisms in the treatment of cancer and other diseases remain to be elucidated. The current research results show that the curative effect of arctigenin is not very significant, and it is difficult to apply in clinical practice. We hope that follow-up studies will map the complete signalling network associated with arctigenin to facilitate future new drug research for potential clinical indications.
- There are few pharmacological experimental studies on arctigenin and its derivatives, and the activities are mainly concentrated in anti-tumour and anti-parasitic activities, and there are few reports in other fields of activity. Moreover, the pharmacological activities of these compounds are mainly concentrated in in vitro experiments, and relatively few in vivo experiments. In addition, many synthetic derivatives have not been studied for activity, which is a pity.
- Pharmacological research on arctigenin and its derivatives preliminarily revealed the development value of arctigenin and its derivatives. In the field of anti-tumour research and development, compounds **145**, **153** and **156** (Figure 21) are the most representative in anti-tumour activity, all exceeding arctigenin. Compound 145 has high yield, strong water solubility, significantly enhanced antitumor activity, and significantly improved oral bioavailability. The inhibition rate was 69.27% ​​at 40 mg/kg. And metabolise in the body to play the role of technical drugs. Compound **145** was developed for arctigenin derivatives with high efficiency, water solubility and high bioavailability provide a new strategy.
- In the field of antiparasitics, mainly including SVCV, *D. intermedius*, IHNV and *Toxoplasma gondii*, arctigenin derivatives all showed strong activity, and compounds **189**, **194**, **196**, **197**, **217** and **243** were the most representative. For anti-SVCV activity, the IC_50_ values ​​of compounds **189** and **196** were 0.077 and 0.095 mg/mL, respectively. The anti-*D. intermedius* activity of compounds **194** and **197**, with EC_50_ values ​​of 2.13 and 2.07 mg/L, respectively. Compound **217** reduces IHNV replication with an IC_50_ of 1.3 μM. Compound **243** exhibited the strongest anti-*Toxoplasma* activity and the lowest cytotoxicity (*Toxoplasma gondii* IC_50_: 17.1 μM; IC_50_: >600.0 μM in HeLa cells; selectivity: 35.09).
- Novel drug delivery systems are effective strategies to improve the water solubility, absorption, distribution, metabolism, excretion (ADME) and toxicity of many drugs^163–164^. The study of arctigenin combined with a new drug delivery system has not been reported yet. The development of novel drug delivery formulations including nanosuspensions, micelles, nanoparticles and nanogels will improve the efficacy, water solubility, bioavailability and targeting properties of arctigenin.
- In most of the above studies, the reported modifications were limited to the hydroxyl groups of arctigenin, and the modifications at the remaining sites were rarely reported. Therefore, future work should be devoted to investigating modifications at other sites. Many natural products are biologically active while providing opportunities for drug discovery in different therapeutic areas. Arctigenin is known to have anti-tumour, anti-inflammatory, anti-leukemia, anti-colitis, anti-virus, vascular protection, hepatoprotective and anti-parasitic biological activities. However, the research on arctigenin derivatives has only focussed on the antitumor and antiparasitic fields. We hope that researchers can explore other pharmacological activities of arctigenin and its derivatives.
- The development of arctigenin-based drug combinations may be a useful strategy, such as combining arctigenin with other anticancer drugs to obtain high anticancer activity, thereby overcoming the insufficient antitumor activity of arctigenin limits.

We believe arctigenin provides a natural product platform for drug development for the treatment of cancer and parasitic diseases. So far, this platform provides a good basis for the development of new derivatives that are more potent and more water-soluble than the natural product arctigenin. Arctigenin derivatives may be potential drugs for clinical treatment of human diseases.
